# Bromine in Hospital Gangrene

**Published:** 1863-09

**Authors:** Wm. B. Alley


BUFFALO

### Medical and Surgical Journal

VOL. III. SEPTEMBER, 1863. NO. 2.

### ORIGINAL COMMUNICATIONS.

ART. I.—*Bromine in Hospital Gangrene.*—By Wm. B. Alley, M. D.

Alvin Dibble, aged 19, a private in Company F, 33d Regiment N. Y. V., received a gun-shot wound on the 3d of last May, at the battle of Fredericksburg, fracturing the upper fifth of the right humerus, and seriously injuring the wrist-joint of the same arm. On the following day he had the head of the humerus exsected; four days afterwards he was removed to some hospital in Washington, where, according to his report the wound steadily improved until it was three-fourths healed up, when Hospital Gangrene appeared, and continued until all the granulating surface, the new cicatrix, a part of the flap made at the exsection, and perhaps two inches in width of the integuments below the wound had sloughed out.

On the 30th day of June he arrived at Mr. Alfred Bell's in Nunda, N. Y., when in consultation with Dr. John Gilmore I first saw the case. We found his pulse 120, feeble, yet regular, appetite gone, and bowels loose. We found a deep, ragged, triangular shaped sore, extending from two inches below the outer edge of the coracoid process nearly down to the insertion of the deltoid muscle, and turning across the arm extending inwards and upwards to within three inches of the external end of the clavicle, leaving a sound tongue of integuments and the deltoid between, being an ill-shaped slough, eight or nine inches long, from one-half to three inches wide, with a little healthy granulating surface, and several insensible grey spots in the wound, emitting an offensive odor, discharging a grayish, sero-purulent fluid.

The gray patches were cauterized with nitrate silver, the balance of the sore well washed in lime water and dressed with a cold linseed poultice—a chalk and opium powder given at night. Dr. Gilmore took charge of the case, and gave him a plain, generous diet, egg-nog and vegetable bitters. The sore generally dressed once in an hour with lint and cold water, it being first well cleansed with lime water and then washed with a solution of per mang, potassa ʒi, water one pint, with an occasional linseed or yeast poultice. This treatment had so far improved the case that on the 12th of July I found his pulse 85, bowels nearly regular, appetite good and strong, skin natural, one-third of the wound nearly cicatrized, the whole sore filled with healthy granulations, and the patient able to sit up and walk about his room, and out into the garden.

Four days afterwards I was advised by Dr, Gilmore that a small grayish spot had appeared in the wound.

On the 18th of July I again saw the case; found his appetite failing, pulse 100, some fever and diarrhœa; a part of the granulating surface and a portion of the integuments at the lower edge of the wound had commenced to slough out, a grayish yellow pus adhered in patches to the granulating surface, and in the upper part of the sore there was a small opening; the granulations around it looked pale and flabby, and were nearly insensible to the touch. On introducing a probe I discovered a loose fragment of bone two inches below the shoulder-joint.

Sunday, July 19th, saw the case with Dr. A. C. Campbell of Mount Morris, and Drs. Gilmore and Upson of Nunda. No change except a stringy, gray matter adhered to a small place in the center, a little below the spiculum of bone found yesterday; all deemed amputation unnecessary. Dr. Campbell advised a plaster, composed of common plaster 100 parts to 8 parts of kerosene oil, with sufficient sweet oil to form a proper paste.

20th.—No real improvement, pulse 105 per minute; the opening in the upper part of wound probed day before yesterday discharges more, and its edges looked gray and flabby. Linseed poultices directed for the night.

21st.—Pulse 115, tongue covered with a dusky white fur, appetite gone, pain and redness about the wound increasing, the odor more offensive, the sore is sloughing out more, a stringy gray pus adheres to several parts of the sore, and a slightly greenish fluid issues from the opening that connects with the fragment of bone. We continued the usual tonic and sustaining treatment; cauterized all the gray spots, removed all the sloughing parts, washed the wound in castile soap suds, and then with lime water, wetting the sloughing portions with whisky; and covering the whole wound with a yeast and charcoal poultice; wound to be dressed every two hours; and a cloth wet with a wash of acetate of lead, laudanum and cold water to be constantly applied to the inflamed parts about the wound.

Wednesday, July 22d.—No improvement, pulse 120, more fever; most of the new granulations had sloughed out, the opening connected with the bone growing larger, the discharge increasing in quantity and offensiveness.

At 11 o'clock A. M. a consultation was had; Drs. Gilmore, Upson, Warner, Meacham, Booth and myself, present. Opinion unanimous, that immediate amputation of the arm afforded him the only chance of final recovery.

At 2½ o'clock P. M. with the assistance of the above named gentlemen I removed his arm at the shoulder-joint, loosing less than ten ounces of blood. Found gangrenous appearances far more extensive than was anticipated. I found the textures near the joint soft and tender. I removed muscular tissue of a pale yellow color, as far back as the neck of the scapula, and in front to the external end of the clavicle; also quite a piece of the subscapularis muscle from under the coracoid process. The dissection was continued as long as we dare keep him under anæsthetic influence, and we hoped that all the diseased tissues were removed; had to use about one square inch of the new cicatrix in order to get sufficient integument to close the wound. Used two parts of Squibb's ether and one part chloroform as an anæsthetic; it operated very kindly; he rallied slowly, sweating profusely for six hours after operation. Pulse 130, at 6 P. M.

On examination of the removed arm we found a fractured, loose, thin rim of bone still surrounding two-thirds the upper and sawed end of the humerus, one-fourth of an inch long, which the surgeon who exsected the head had sawed off, but failed to discover and remove. This fragment being left is, perhaps, why the operation was not successful, terminating as it has. The sawed end of the humerus was rough and diseased for an inch below; all the tissues surrounding its upper third were gangrenous being soft, tender and nearly disorganized, and all the muscles were of a, pale yellow color, as low as the middle of the arm, and portions of them still lower. There were no nerves or arteries injured by the former operation.

At 10 o'clock P. M. pulse 135, and feeble, and the skin dry. At 12 M. he vomited, apparently all he had taken into his stomach for the past 16 hours, and he loathed everything nourishing; gave him 30 drops aromatic spirits ammonia, followed in half an hour with morphine and brandy; after which he rested better.

23d inst. 6 o'clock A. M. Pulse 130; skin dry; took some toast and tea, but did not relish it. 2 o'clock P. M., pulse 125, complains of pain in the arm. 9 P. M., pulse 120, bowels moved freely.

24th, 9 A. M. Rested considerably; pulse 105, skin more natural, appetite improving, wound begins to suppurate. 9 o'clock P. M., pulse 110, skin dry.

25th, at 9 o'clock A. M. Pulse 108; at evening 118; had three stools since noon.

26th, at 8 A. M. Pulse 125; complains of headache and chilly sensations; wound discharges profusely; about an inch of the upper part of wound healed by first intention; yet a part of the cicatrix used as a flap was flabby and insensible; removed the flabby portion of the cicatrix, cauterized the freshly incised parts, syringed the cavity with lime water, dressing it with a yeast and charcoal poultice, to be changed every two hours; the other parts of the wound to be dressed with lint and cold water every hour. Gave ℥ss wheat whiskey once in three hours, and mutton broth or toast as often. 3 o'clock P. M., pulse 130, tongue moist, covered with a yellowish fur, diarrhœa worse, intellect clear, breath offensive, odor from wound bad; penciled the cavity with a solution of creosote, directed ten drops mur. tinct. of iron every six hours, whisky between, and chalk and opium powders after each evacuation until the diarrhœa abated.

Having read in the *American Medical Times* Dr. R. L. Stanford's article on Bromine in Hospital Gangrene, I resolved to try it, if I could procure the bromine.

27th, 8 o'clock A. M. Rested badly, countenance anxious, pulse 140, diarrhœa better, appetite gone, complains of severe pain in and around the wound, one small gray spot in one corner of wound; that part of the flap made from the newly cicatrized granulations was of a dark purple color emitting a foul odor; beef tea to be tried during the day. 9 o'clock P. M., patient uneasy, headache, pains about the shoulder, pulse 144, tongue moist and coated with a dirty yellow fur; loathed the beef tea, diarrhœa worse, the cavity enlarging, and discharging profusely; gray spot extending, the purple sloughing cicatrix discharged a greenish yellow, offensive fluid; without doubt hospital gangrene had appeared.

Bromine ordered is on hand. Dissected away all the diseased cicatrix, and gray insensible spots, down to red bleeding tissues; cleansed the cavity thoroughly, and dried with warm dry lint, and applied fresh pure bromine to every part supposed to have any gangrenous appearance. The cavity to be dressed every hour, with a fresh cinchona poultice; the other part of the sore with lint and cold water once in half an hour, and tinct. of arnica applied about the shoulder. A large chalk, kino and opium powder given; stimulants as usual.

28th, 8 o'clock A. M. Pulse 140, mind lucid, rested more quiet, bromine destroyed all the bad odor; no appetite, diarrhœa less, wound dark colored and discharging largely. 3 o'clock P. M., pulse 140, but stronger; urine scanty and high colored; color of cavity improved, but the odor is offensive; applied the bromine again; the ligature from the posterior circumflex artery came away. Diet; soft boiled eggs, tea and toast. Treatment; vegetable and aromatic bitters in whisky every eight hours, after eating; ten drops muri. tinct. iron every six hours, and egg-nog or mutton broth between.

29th, 7 o'clock A. M. Countenance better, pulse 136, pain and soreness about the wound less. Either the bromine or the fluids from the sore had created a severe erythema of the skin, extending several inches over the scapula and down the back, although great pains had been taken to prevent both from coming in contact with the sound skin. Bromine applied again, and the whole cavity and exposed surface washed with a solution of potass, per mang. and dressed with lint and cold water every hour. Same treatment continued. 1 o'clock P. M,, pulse the same. At 8 o'clock P. M., pulse 134, appetite improving, had one natural stool; bromine has eaten off the visible part of the ligature of the brachial and perhaps the anterior circumflex arteries; there is less odor, less discharge, and some healthy granulations appearing.

30th, at 8 o'clock. Pulse 128; at night 122, bowels and appetite much improved.

31st. Diarrhœa again, but pulse 112,

August 1st. Same treatment continued. Bromine discontinued, except occasionally a tincture of it to destroy any odor that was present. Pulse 106; all things mending.

2d. Pulse 104.

4th. Pulse 100, appetite good, discharge ceasing.

6th. Pulse 96.

8th. Pulse 90 to 100; bread, milk and baked apples added to diet list; sore dressed with linseed oil ointment.

11th. Walked into the parlor alone; appetite excellent, bowels regular, allowed to eat with family.

15th, Rode out.

18th. Rode one-third of a mile and walked into my office to make me a visit. Pulse 100; system in fine condition; wound, four-fifths cicatrized.

I feel that bromine arrested gangrene in this case, being the main arm, that snatched this young man from the jaws of death. Still, he in fact owes his life to Mr. and Mrs. Bell. They gave him their best rooms and undivided attention, doing all that money and good nursing could do. No patient ever had better care; had it been otherwise he must have gone down. It is true I used other remedies; among them was the solution of potass, per mang., recommended by Surgeon-General Hammond, but I failed to see that it had any more effect than the cold water dressing.

As there are so many poor soldiers in our country liable just now to hospital gangrene, and as our text books furnish us with no specifics, I have reported the foregoing case, hoping it might tend to give others more confidence to try Dr. M. Goldsmith's remedy.

I wish you would give to the public your confidence in and knowledge of the use, of pure bromine in hospital gangrene. Soldiers are frequently coming home with what is supposed to be hospital gangrene, and I have heard of its causing several deaths within a few months.

